# Anti-Tumoral and Anti-Angiogenic Effects of Low-Diluted *Phenacetinum* on Melanoma

**DOI:** 10.3389/fonc.2021.597503

**Published:** 2021-03-03

**Authors:** Camille Fuselier, Sandrine Quemener, Eleonore Dufay, Camille Bour, Camille Boulagnon-Rombi, Nicole Bouland, El-Hadi Djermoune, Jérôme Devy, Laurent Martiny, Christophe Schneider

**Affiliations:** ^1^Université de Reims-Champagne-Ardenne, CNRS UMR 7369 (Matrice Extracellulaire et Dynamique Cellulaire, MEDyC), Reims, France; ^2^Université de Lille, Institut Pasteur de Lille, U1011 INSERM, Lille, France; ^3^Centre Hospitalier et Université de Reims Champagne-Ardenne, laboratoire de Biopathologie, Reims, France; ^4^Université de Reims Champagne-Ardenne, laboratoire d’Anatomie Pathologie, Reims, France; ^5^Université de Lorraine, CRAN, UMR CNRS 7039, Nancy, France

**Keywords:** melanoma, cancer, angiogenesis, phenacetin, *in vivo*, homeopathy, tumor-associated macrophages, metastasis

## Abstract

Melanoma is the most aggressive form of skin cancer and the most rapidly expanding cancer in terms of worldwide incidence. If primary cutaneous melanoma is mostly treated with a curative wide local excision, malignant melanoma has a poor prognosis and needs other therapeutic approaches. Angiogenesis is a normal physiological process essential in growth and development, but it also plays a crucial role in crossing from benign to advanced state in cancer. In melanoma progression, angiogenesis is widely involved during the vertical growth phase. Currently, no anti-angiogenic agents are efficient on their own, and combination of treatments will probably be the key to success. In the past, phenacetin was used as an analgesic to relieve pain, causing side effects at large dose and tumor-inducing in humans and animals. By contrast, *Phenacetinum* low-dilution is often used in skin febrile exanthema, patches profusely scattered on limbs, headache, or flushed face without side effects. Herein are described the *in vitro*, *in vivo*, and *ex vivo* anti-angiogenic and anti-tumoral potentials of *Phenacetinum* low-dilution in a B16F1 tumor model and endothelial cells. We demonstrate that low-diluted *Phenacetinum* inhibits *in vivo* tumor growth and tumor vascularization and thus increases the survival time of B16F1 melanoma induced-C57BL/6 mice. Moreover, *Phenacetinum* modulates the lung metastasis in a B16F10 induced model. *Ex vivo* and *in vitro*, we evidence that low-diluted *Phenacetinum* inhibits the migration and the recruitment of endothelial cells and leads to an imbalance in the pro-tumoral macrophages and to a structural malformation of the vascular network. All together these results demonstrate highly hopeful anti-tumoral, anti-metastatic, and anti-angiogenic effects of *Phenacetinum* low-dilution on melanoma. Continued studies are needed to preclinically validate *Phenacetinum* low-dilution as a complementary or therapeutic strategy for melanoma treatment.

## Introduction

Melanoma is an aggressive and metastatic skin tumor, defined as the 12th most common cancer in the world. Epidemiological data demonstrated that it represents only 4% of dermatological cancers, but it has become responsible for 80% of deaths in the case of metastatic progression (1, 2). A continued increase in the number of new cases has been observed for the last 40 years among white populations of different countries, and it is estimated annually between 3% and 7% depending on the country (1, 3). Thus, cutaneous melanoma is a disease of multifactorial causality and the survival of patients decreases depending on its stage of advancement (4). Indeed, primary tumor is partly developed by an excessive proliferation of mutated melanocytes, which can be followed by a metastatic progression through a complex mechanism such as cell detachment, cell migration, and cell invasion (5). So, while primary cutaneous melanoma is mostly treated by means of curative wide local excision (6), in the case of an advanced-stage melanoma, other therapeutic strategies must be applied. Among them, chemotherapy (vemurafenib or dacarbazine), immunotherapy that represents a promising form of treatment targeting immune check points (PD1, Programmed Cell Death; CTLA, Cytotoxic T-Lymphocyte-Associated Protein), targeted therapy, or cell therapy meant to boost T lymphocytes activity and radiotherapy are used alone or in combination so as to increase their efficacy (7). However, these treatments are generally considered ineffective, leading frequently to the development of chronic side effects, toxicity for patients, and finally to therapeutic failures (6, 7). Moreover, the plasticity of the tumor microenvironment can also contribute to treatment resistance and pathological development associated with fatal issues (8).

Angiogenesis described for the first time in melanoma by Warren in 1966 (9) plays a crucial role in cutaneous melanoma progression since it’s fundamentally involved in the vertical growth phase (10, 11). Indeed, the rapid proliferation of tumor cells increases their demand for oxygen and nutrients, which leads to local hypoxia and subsequent signaling cascades to offset its effects. Therefore, tumor angiogenesis is characterized by the formation and recruitment of new blood vessels from preexisting vessels to meet the physiological needs of the tumor (10). In the microenvironment of melanoma, tumor cells, tumor-infiltrating inflammatory cells, and endothelial cells are all involved in the production of growth factors and cytokines, able to stimulate (VEGF, FGF…) or inhibit (TIMP…) the angiogenic response (8, 12). As suggested by Folkman in 1971, this process has been therefore a particularly attractive therapeutic target for many years as an anti-cancer strategy (10, 11, 13). Thus, toxicity issues, side effects, and individual resistance found in the most available anti-angiogenic treatments should be considered to establish original combinatorial strategies. That is why it is essential to develop complementary and innovative therapeutic strategies in order to improve the effectiveness of existing treatments.

In this way, the use of complementary and alternative medicines (CAM), including homeopathy, appeared over the last 3 decades. Oncologists prescribe not only anticancer treatments but also included CAM, in particular homeopathy as integrative therapy (14). Samuel Hahnemann adopted a new way of thinking and used a specific form of remedy preparation, the stepwise dilution and a standardized succession called “potentization” (15, 16). Currently, homeopathic preparations are mainly used for improving the cancer patients’ quality of life, but further studies have raised additional valuable effects in cancer cell lines, *in vitro* and *in vivo* (17, 18). The “Canova method” composed of several homeopathic dilutions is reported for activating the immune system *via* macrophages, and *Lycopodium clavatum* has the capacity to induce apoptosis in HeLa cancer cells (19, 20). Moreover, *Ruta graveolens* 200c and *Hydrastis Canadensis* 200c have significantly reduced the solid tumor volume of carcinoma and lymphoma in transplanted mice (21). Considering all these studies and despite a context that tends to question the existence of any effect related to homeopathic treatments, we have already evidenced the impact of low homeopathic dilutions of phenacetine (*Phenacetinum* 4CH) on murine melanoma progression (22). The phenacetine molecule is a chemical aromatic organic compound initially used as an analgesic or anti-pyretic. However, phenacetin has exhibited carcinogenicity in renal pelvis and urinary bladder of patients and was withdrawn from the market early in the 1980s (23, 24). However, despite these harmful effects, the use of a substance potentially toxic yet highly diluted (for example, cadmium or arsenic) can lead to an effective reduction of their toxicities *in vitro*/*in vivo* and to improve its benefits (25–27).

Based on this knowledge, understanding the mechanism of low-diluted *Phenacetinum* action through *in vitro* study and preclinical evaluation by *in vivo* tumor transplantation remains an effective method of demonstrating its efficacy in cancer. Hence, herein we have transposed the promising effects of *Phenacetinum* 4CH characterized *in vitro* (22) on through *in vivo* studies to evaluate the potential anti-neoplastic and/or anti-angiogenic effect(s) of this homeopathic treatment on primary melanoma tumor growth and vascularization, as well as on lung metastasis and in angiogenesis Matrigel**^®^** plug assays by DCE-RMI. Given the complexity of the process studied, the combination of several tests is necessary to understand the cellular mechanisms involved during angiogenesis. Henceforth, we used *ex vivo* (IHC, aortic ring assays) analyses and *in vitro* angiogenesis assays (tubulogenesis).

## Materials and Methods

### Cell Culture, Reagents, and Antibodies

B16F1 cancer cell lines were obtained from the American Type Culture Collection (ATCC) and maintained in RPMI-1640 medium (Gibco, Life Technologies, Saint-Aubin, France) supplemented with 10% fetal bovine serum (FBS, ATCC) in standard conditions (5% CO_2_, 37°C). Human Umbilical Vein Endothelial Cells (HUVEC) were purchased from Promocell (Heidelberg, Germany). Cells were cultured in ready-to-use endothelial cell growth medium (ECGM2) (Lonza, France). Matrigel^®^ was purchased from Dutscher (France). All cell lines were used at low passage number and were mycoplasma free (MycoAlert; Lonza). Anti-CD31 antibody (#557355) used for immunohistochemistry was from BD Pharmingen, and anti-CD163 (#ab182422) and anti-CD68 (#ab125212) were from Abcam (France). Biotinylated anti-rat IgG (#BA-4001) used as secondary antibody was from Vector Laboratories (Abcys) and rabbit detection kit from Abcam (#ab64264). Homeopathic dilution, *Phenacetinum* 4CH, was obtained from BOIRON laboratories (Messimy, France): 1 g of Phenacetine was first diluted in 99 ml d’H_2_O and dynamized or succussed to obtain the first dilution. This procedure was repeated with dynamization four times to obtain 4 CH (1x10^-8^) with final concentration at 0.563 nM.

### Animals

Eight-week-old female C57BL/6 mice (average body weight, 18–20 g) were purchased from Charles River Labs (France). Animals were kept in a room with constant temperature and humidity, and food and water were given *ad libitum*. Mice were acclimatized to our laboratory conditions for 1 week before starting the experiments. The *in vivo* experiments were conducted in conformity with institutional ethical guidelines of the University of Reims Champagne-Ardenne (CEEA-RCA n°56) and the CNRS (Centre National de la Recherche Scientifique), which comply with international laws and policies. The ethics committee approved the protocol “Influence of 3 homeopathic drugs on tumoral development in a melanoma syngenic mice model” under the 8135v3 number. All procedures performed in studies involving animals were in accordance with the ethical standards of the institution or practice at which the studies were conducted.

### Subcutaneous *In Vivo* Models

For the subcutaneous tumor model, suspensions of B16F1 cells (2.5 × 10^5^ cells in 100 μl RPMI-1640 medium) were subcutaneously injected into the left flank of different randomized series of syngeneic C57BL/6 mice (n=20 per group), as previously described (28). Intraperitoneal administrations of *Phenacetinum* 4CH (5 ml/kg) or controls (H_2_O 100 µl/mouse) were performed in double-blind each day. Tumor volume was determined according to v = (AxB^2^)/2, where A denotes the largest dimension of the tumor and B represents the smallest dimension (29). At day 14, mice were sacrificed, and tumors were surgically extracted, weighed, and fixed in 4% paraformaldehyde or frozen for immunohistochemistry. Mice were also weighed every 2–3 days and daily checked for any modification in their behavior. Loss of 15% body weight was an indication for euthanasia as well as severe tumor necrosis, as it was sometimes associated with skin ulceration that would result in loss of body fluid and/or infection. The survival time was determinate until animals died or reach a maximum of 1 cm^3^ tumor volume (or any other limit points previously determined: 15% loss of body weight, prostration of the animal, ataxia).

### Experimental Metastasis Model

For the induced lungs metastasis model, suspensions of B16F10 cells (1 × 10^5^ cells in 100 μl RPMI-1640 medium) were injected in the lateral tail vein of different randomized series of syngeneic C57BL/6 mice (n=10 per group), as previously described (30). Intraperitoneal administrations of *Phenacetinum* 4CH (5 ml/kg) or controls (H_2_O 100 µl/mouse) were performed in double-blind each day. On day 14, mice were imaged by µCT. On day 21, DCE-RMI was performed. Mice were sacrificed and lungs were collected for quantification of metastases and fixed in 4% paraformaldehyde for histopathological analyses. Mice were weighed every 2–3 days and daily checked for any modification in their behavior. Loss of 15% body weight and respiratory distress were indications for euthanasia (or any other limit points previously determined: prostration of the animal or ataxia).

### Histopathological Analysis of Tumors

Histological analysis of formaldehyde-fixed and paraffin-embedded melanoma tumors were performed on hemalun, phloxin, and saffron (HPS) stained 3 μm thick sections prepared using routine histological methods. To assess tumor-associated microvessels density (MVD), CD31 (BD Pharmingen, USA) immunostaining was performed on 5 μm thick cryosections using biotin-labeled secondary antibody and streptavidin-HRP AEC detection system (Microm Microtech, Francheville, France), followed by hematein counter-coloration. Negative controls were done omitting the primary antibody. The necrotic part relative to total tumor surface for each tumor slice as well as MVD were quantified using ImageJ software. Also, 3 µm paraffin thick sections were used for CD163 and CD68 immunostaining. CD163 and CD68 expression were expressed as the average number of CD163+ and CD68+ cells in 10 high-power fields (HPF, x100 or x200) in IHC sections, selected randomly.

### μCT Imaging of Mice

Animals were anesthetized by isoflurane (Forene, Abbott France, Rungis, France) inhalation (3–5% for induction and 2–3% for maintenance) and then after placed in a warm imaging bed (Minerve, Esternay, France) allowing the maintenance of isoflurane anesthesia. CT images were acquired on a dedicated small animal μCT scanner (Skyscan1076, Bruker, Kontich, Belgium) while continuously rotating the camera by 180° with the following parameters: 80 kV, 0.5 mm Al filter, 120 μA source current, 35 μm isotropic resolution, 230 ms exposure time, 2 projection images per 0.5° rotation step, and a prospective respiratory synchronization. The projections were reconstructed using a filtered backprojection algorithm using Skyscan software (NRecon, Skyscan). For tumor angiography analysis, an alkaline earth-based nanoparticulate contrast agent (Viscover ExiTron nano 12000, Miltenyi Biotec, Paris, France) was injected in the mouse tail vein. Mice were imaged during the next 30 min following injection, a period during which no reduction in contrast was observed (31). Analysis of 3D reconstructed images and quantification of the vascular network were performed using Amira 6.5 software (Thermofisher, USA).

### MRI Imaging

MR images were acquired using a mouse body coil. Temperature was maintained by a heating air flow built into the animal bed system. Respiration was monitored throughout the entire scan. For Matrigel**^®^** plug assay, images were acquired using a three-dimensional (3D) T2-weighted fast spin echo sequence (FSE) coronal T2-weighted fast spin echo sequence (FSE) with echo time (TE)/repetition time (TR) = 68/5000 ms, 60 mm x 60 mm field of view, 1 mm slice thickness, and 512 x 240 matrix (with 2x oversampling in read direction) zero-padded to 512 x 256 during reconstruction (resolution = 0.234 mm × 0.25 mm), echo train length = 8, number of averages = 1, effective receiver bandwidth ([BW] = 20 kHz); a transverse T2-weighted fast spin echo sequence (FSE) with echo time (TE)/repetition time (TR) = 68/5000 ms, 40 mm x 40 mm field of view, 1 mm slice thickness, and 256 x 240 matrix zero-padded to 256 x 256 during reconstruction (resolution = 0.156 mm × 0.167 mm), echo train length = 8, number of averages = 2, receiver bandwidth ([BW] = 20 kHz); Matrigel^®^ plug perfusion was measured in DCE-MRI (TR = 60ms, flip angle = 25° and 105 experiments) by intravenous bolus injection of Clariscan™ (gadoteric acid, GE Healthcare), 100 µl, 0.2 mmol/Kg at experiments 6/105.The total imaging time was ~40 minutes. Intensity analyses were performed after 4D reconstruction with OsiriX software (Pixmeo, Swiss) using ROI (Region Of Interest) enhancement plugin. The wash-in-rate values were calculated during the first 120 s after Clariscan™ injection. For lung metastases, images were acquired using a fast low angle shot magnetic resonance imaging (FLASH MRI), flip angle 20°, 10 averages, 256 views, 35 mm x 35 mm field of view, 0.8 mm slice thickness, number of averages = 10.

### Mouse Aortic Ring Assay

Thoracic aortae were excised from C57BL/6 mice and peri-adventitial fibro-adipose tissues were removed. Aortae were then cut into 1 mm rings, washed and transferred to 48-well tissue culture plates coated with 100 μl per well of Matrigel**^®^**. Explants were then overlaid with additional 100 μl of Matrigel**^®^**. After polymerization, endothelial growth medium was added supplemented with 5% (v/v) *Phenacetinum* 4CH or vehicle and then renewed every 3 days. Digital images were taken for quantitative analysis of vascular endothelial outgrowth using ImageJ software and a macro developed under Matlab by El-Hadi DJERMOUNE (32).

### Matrigel^®^ Plug Assay

C57BL/6 mice (n=7 per group) were anesthetized and injected subcutaneously with 400 μl Matrigel™ (growth factor reduced) containing 100 ng/ml VEGF, 350 ng/ml FGF-2, and 50 U/ml heparin. Negative control mice were injected with Matrigel**^®^** without any additional growth factor. Control group (H_2_O 100 µl/mouse) and experimental group (*Phenacetinum* 4CH 5 ml/kg) were injected intraperitoneally every 2 days until J14. Mice were then anesthetized using isoflurane, cannulated *via* the lateral tail vein for delivery of gadolinium, and imaged using MRI (3T, MR solutions, Guildford, UK).

### Tube Formation Assay

Pre-chilled µ-Slide Angiogenesis plates (Ibidi, 81506) were coated with 10 µl**^®^** Matrigel (10 mg/ml) and were allowed to polymerize for at least 30 min before use. HUVEC (P2 to P5, 5 × 10^4^) were then seeded on Matrigel**^®^** layer in the endothelial basal medium supplemented with 1 % (*v/v*) FCS, 1 μg/ml ascorbic acid, and 0.2 μg/ml hydrocortisone, with or without (negative control) 10 ng/ml VEGF into each well in triplicate. After 4 h to 6  h at 37 °C, cells were imaged at × 4 magnification on a Nikon eclipse 300 inverted microscope. Total network length, node number, junction number, and mesh number were analyzed using Gilles Carpentier’s Angiogenesis Analyzer for ImageJ plugin (33). The results are the means of random fields in 3 replicates.

### Cell Viability Assay

The cells were seeded at a density of 5 × 10^3^ cells/well in a 96-well culture plate. Homeopathic drugs are administrated in wells for 5% and stopped after 24 h of incubation (5% CO2, 37°C). For that, 20 µl of MTT solution at [5 mg/ml] were added in wells and incubated during 3 h (5% CO2, 37°C). The media were gently removed and 100 µl of DMSO were added to dissolve formazan crystals. MTT reduction was quantified by measuring the light absorbance at 570 nm using the reader microplate (TECAN, Infinite). Each experiment was repeated three times.

### Transwell Migration Assay

A 24-well Transwell chamber (Greiner Bio-One, Dutscher, France) with an 8-*μ*m pore PET membrane was used to perform the migration assay and coated with collagen I (R&D System) at 10 µg/ml. The lower chamber was filled with 600 µl RPMI 1640 B16F1 or B16F10 conditioned medium (made from B16F1 or B16F10 control and *Phenacetinum* 4CH-treated cell line). Then, 5 × 10^5^ HUVEC cells suspension were added into the insert. The cells were allowed to migrate at 37°C with 5% CO2 over 18 h. The inserts were washed in PBS and fixed with methanol for 15 min. Non-migrating cells were removed from the upper surface of the inserts by gently scrubbing with a cotton-tipped swab. Each PET membrane was cut and stained with mounting medium DAPI (ProLong Gold DAPI, ThermoFischer), between blades and slats. For counting, 10 pictures were taken per membrane, and each condition was made in duplicate. Experiments were performed in triplicate.

### Statistical Analysis

GraphPad Prism 8.4 software (GraphPad, La Jolla, CA) was used for all statistical analyses. Each result is representative of at least 3 independent experiments. Data are expressed as the mean +/- SEM. Significance was assessed using Student’s *t* test or One Way ANOVA for *Gaussian distribution of datas* parametric tests and *Mann-Whitney U* test was used for *others (non-parametric test)*. For all tests, statistical significance was assumed when *p* < 0.05 (*).

## Results

### *Phenacetinum* 4CH Inhibits *in Vivo* Tumor Progression in a Mouse Melanoma Model

**Figure 1:**
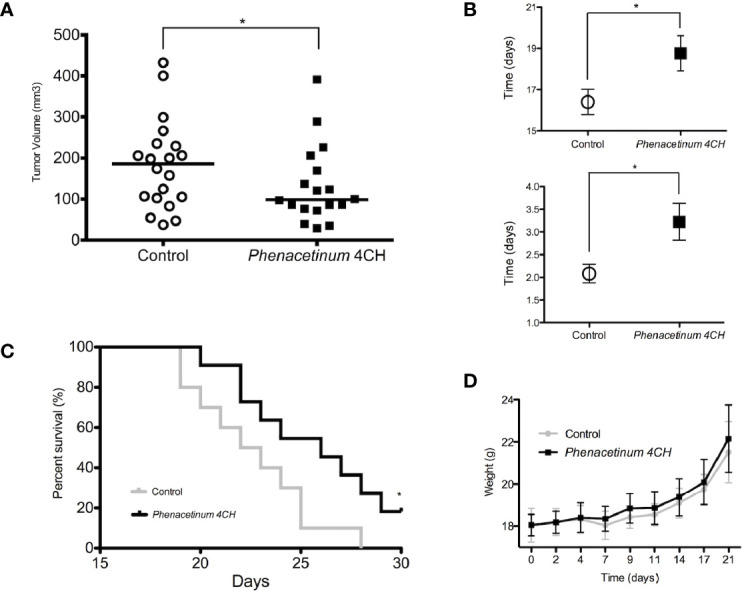
Effect of *Phenacetinum* 4CH on tumor progression, body weight, and survival time 2.5 x 10^5^ melanoma B16F1 cells were subcutaneously injected into the left side of C57BL/6 mice. Control (open circle) or *Phenacetinum* 4CH (filled box) (100 µl) were intraperitoneally injected each day. **(A)** Tumor volume was measured with a caliper as described in Materials and Methods. Graph represents tumor volume measurements at day 14 after melanoma cells injection. *Horizontal lines*, median (*n* = 20, *Mann-Whitney* test). **(B)** Time to reach a tumor volume of 300 mm^3^ (1B high) and doubling tumor volume time (1B low) during the exponential growth phase (days 14 to 17). **(C)** The survival rates of C57Bl/6 mice with B16F1 melanoma xenografts treated with Control (grey line) or *Phenacetinum* 4CH (black line) were recorded and shown as Kaplan-Meir curves. **(D)** Mouse mean weight evolution for control (grey line) and *Phenacetinum* 4CH (black line) groups. (*p < 0.05).

To study the effect of *in vivo Phenacetinum* 4CH, we have implemented a double-blind daily treatment that started on the same day as the inoculation of the tumor cells. B16F1 tumor cells were injected subcutaneously and tumor growth monitoring was done every day from day 7 to the end of the experiment (death of the mice or a 1000 mm^3^ tumor volume). The graphic represented in **Figure 1A** shows subcutaneous tumor volumes measured after 14 post-injection days in the control and the *Phenacetinum* 4CH treated group. A significant decrease of 20% of primary tumor volume can be observed, with a median volume of 85.9 mm^3^ for the mice treated with *Phenacetinum* 4CH compared with a median volume of 107.3 mm^3^ for the control mice. In addition, in the case of the tumor volume being treated by *Phenacetinum* 4CH, delay of the time taken to reach 300 mm^3^ is by 2.4 days (18.8 days) compared to the control condition (16.4 days) (**Figure 1B**, high). Similarly, the doubling time of the tumor volume during the exponential growth phase (between days 14 and 17) is much longer in the mice treated by the homeopathic dilution with a significant benefit of 1.14 days (**Figure 1B**, low). Henceforth, *Phenacetinum* 4CH treatment (**Figure 1C**) extends the lifespan of mice by 3.5 days (median survival time of 26 days) compared to that of the control mice (median survival time 22.5 days). Interestingly, no side effects were observed on the weight of the mice (**Figure 1D**) or on the cell morphology of other mice organs (**Figure S1**). Overall, these results suggest that *Phenacetinum* 4CH treatment may reduce tumor onset time and *in situ* growth of B16F1 melanoma cells in C57BL/6 mice.

### *Phenacetinum* 4CH Treatment Disturbs Melanoma Tumor Vascularization *in Vivo*

**Figure 2:**
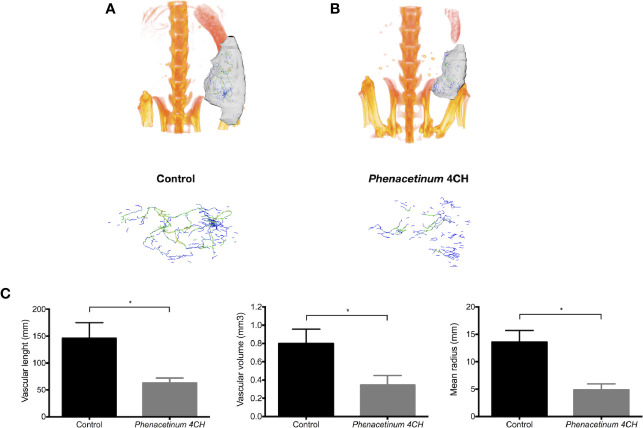
Effect of *Phenacetinum* 4CH on *in vivo* tumor vascularization. **(A, B)** are representative tridimensional reconstructions of tumors at day 14 after µCT reconstruction. Melanoma tumors were segmented, and the associated vascular networks were traced using filament editor of Amira 6.5 software. Color-coded representation of tumor-associated blood vessel network depended on structure thickness (Amira 6.5 software). Thin structures are represented in blue, which changes to green and yellow when the vessel diameter increases. **(C)** is quantification of tumor-associated blood vessel mean length, volume, and radius using Amira 6.5 software. Data are expressed as the mean +/- SEM (*p < 0.05).

To establish whether *Phenacetinum* 4CH-induced volume tumor reduction could be due to the modulation of the tumor angiogenesis, we performed a longitudinal follow-up of the B16F1 melanoma allografts using μCT (**Figure 2**). On day 14, the tumor vasculature of the control mice was predominantly present inside the tumors, but was also apparent at their periphery, which turned out as a mature network (green and yellow vascular segments, **Figure 2A**, supplemental data). Interestingly, thanks to the *Phenacetinum* 4CH treatment (**Figure 2B**, supplemental data), the tumor vascular system seemed to be limited mainly to the periphery of the tumor and composed mostly of blue vascular segments exhibiting especially small diameters, indicative in this case of a largely anarchic and discontinuous network. Consistently, the segmentation and quantification of the tumor-associated vascular network confirmed that *Phenacetinum* 4CH induced a significant 2.3-fold reduction in mean blood vessel volume (mean vascular volume of 0.8 mm^3^ for control mice and 0.34 mm^3^ for the treated mice) (**Figure 2C** middle). The treatment reduced also significantly the length of the blood vessels by 57% (mean length 146 mm in the control case versus 63 mm in the treated case) (**Figure 2C** left). Finally, the mean diameter of the blood vessels decreased by 64% with *Phenacetinum* 4CH (mean diameter of 13.6 mm for control mice and 4.9 mm for treated mice) (**Figure 2C** right). Taken all together, these results demonstrate that *Phenacetinum* 4CH alters the quality and structure of the melanoma tumor-associated blood vessels in mice (videos in supplemental data).

### *Phenacetinum* 4CH Disrupts the Vascularization of Matrigel^®^ Plugs

**Figure 3:**
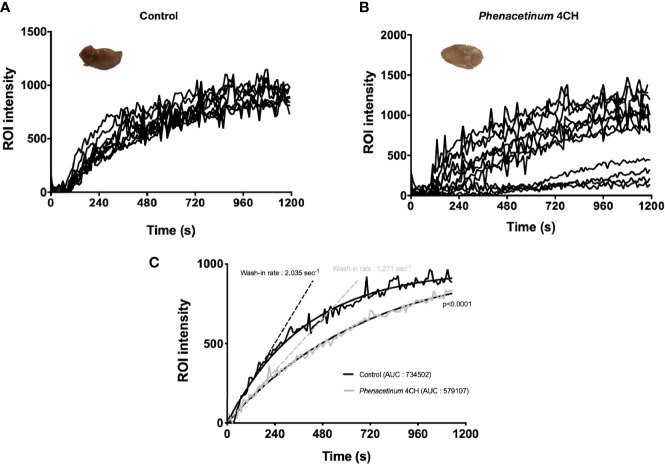
Inhibition of Matrigel^®^ plug angiogenesis by *Phenacetinum* 4CH. FGF- and VEGF-induced Matrigel^®^ plug angiogenesis assay was performed on the back on C57Bl/6 mice. Mice were perfused, injected with 0.1 mmol/kg of Galodidium, and DCE-MRI analyzed. **(A)** Matrigel^®^ plug’ ROI intensity of control mice during perfusion. **(B)** Matrigel^®^ plug’ ROI intensity of *Phenacetinum* 4CH mice during perfusion. **(C)** Pool of control (black) and *Phenacetinum* 4CH (gray) ROI intensity curves and wash-in rate tangents.

To strengthen the observed anti-angiogenic potential of *Phenacetinum* 4CH, we studied its effects *in vivo* on Matrigel^®^ plug assay in mice using a perfusion DCE-MRI approach. After Matrigel^®^ plug injection, mice were treated with *Phenacetinum* 4CH or vehicle every 2 days until the end of the experiment. A dynamic contrast enhancement sequence was performed on mice after a perfusion of an MRI contrast agent in order to quantify the Matrigel^®^ plug perfusion. The results are presented in **Figure 3**. All control mice exhibit a regular vascular network with outstanding reproducibility in the intensity of the signal perfusion (**Figure 3A**). When mice are treated with *Phenacetinum* 4CH, the perfusion signal appears heterogeneous and disrupted (**Figure 3B**). **Figure 3C** exhibits the pool of curves from **Figures 3A, B**. A significant difference (p<0.0001) in Matrigel^®^ plug perfusion can be observed between control and *Phenacetinum* 4CH-treated mice. Control mice have a wash-in rate 40% significantly higher compared to treatment (2,035 sec^-1^ vs 1,271 sec^-1^). Moreover, areas under the curve (AUC) show lowest total perfusion rate when mice were treated with *Phenacetinum* 4CH (-20%). Herein, this significant inhibitory effect of *Phenacetinum* 4CH on Matrigel^®^ plug perfusion suggests a dysfunction of the blood vascular system.

### *Phenacetinum* 4CH Treatment Induces Necrosis

**Figure 4:**
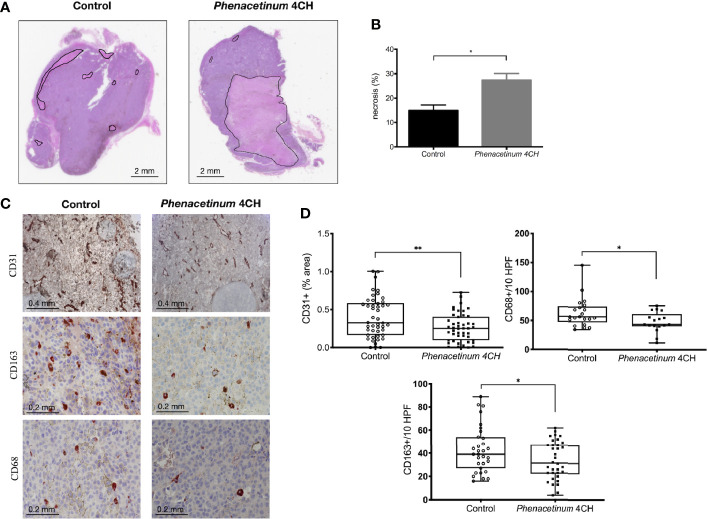
Effect of *Phenacetinum* 4CH on *ex vivo* necrosis, CD31, CD163, and CD68 expression. Photographs 4A and 4C show representative tumor sections from control or *Phenacetinum* 4CH treated mice. **(A)** HPS staining of tumor sections (left panel: control, right panel: *Phenacetinum* 4CH) showing tumor necrosis (area delimited by continuous line). **(B)** Semi-quantitative analysis with Image J software of necrosis area (% of total surface). **(C)** IHC staining in control tumor (left column) and *Phenacetinum*-treated tumor (right column) using anti-CD31, CD163, and CD68 antibodies. **(D)** Semi-quantitative with image J software analysis of CD31 staining (% of total surface) and expression of CD163+ and CD68+ cells (number/10 High-Power Fields). Data are expressed as the mean +/- SEM (*p < 0.05, **p < 0.01).

Following the interesting results observed *in vivo*, we recovered the tumors to perform immunohistochemical complementary studies (**Figure 4**). The tumor tissue consists of tumor cells and supportive tissue (or tumor stroma), itself consisting of cells and extracellular matrix network in which the tumor vasculature is located. The first part of tumors was used to perform HPS staining to quantify tumor necrosis (identified as a light pink, **Figure 4A**). Visually, control tumors show fewer necrotic regions compared to tumors treated with *Phenacetinum* 4CH (intra tumor localization, black line delimited) (**Figure 4A**). Indeed, the quantification of these necrotic foci using image J software shows that the control tumors have a 14.9% necrosis area whereas the treated tumors total 27.6% (**Figure 4B**). Thus, *Phenacetinum* 4CH induces a significant 1.8-fold increase of necrosis in melanoma tumors.

### CD31, CD163, and CD68 Expression in *Phenacetinum* 4CH-Treated Tumor Mice

In addition to necrosis results, immunostaining of tumor sections with an anti-CD31 antibody allowed us to specify the state of vascularization of tumors (**Figure 4C**). Immunological staining reveals that the control tumors have large vascularized areas (brown markings) unlike tumors treated with *Phenacetinum* 4CH. These observations were confirmed by quantification of the CD31-labeled areas through image J software, since *Phenacetinum* 4CH decreased significantly tumor neovascularization by 1.5-fold (mean percentage at 0.45 for control tumors versus 0.30 for treated tumors, **Figure 4D**). These different analyses bear the idea that an impairment of the tumor vasculature by *Phenacetinum* 4CH could lead to a stronger necrosis in tumor-treated mice, resulting in the tumor volumes reduction observed previously. As the tumor microenvironment is an important player in tumor progression, we investigated immunohistological expression of TAM (tumor-associated macrophages) using CD163 and CD68 markers in tumors. As shown in **Figures 4C, D**, the expression profile for both CD163+ and CD68+ cells is altered under *Phenacetinum* 4CH treatment. The percentage of CD68+ and CD163+ TAMs is lower in *Phenacetinum* 4CH-treated mice (-21% for CD163 and -24% for CD68) comparative to the control group. These expressions are correlated with inhibition of tumor size shown in **Figure 1**. These observations underlined the significance of TAMs in *Phenacetinum* 4CH anti-angiogenic effects.

### *Phenacetinum* 4CH Inhibits VEGF-Induced Neoangiogenesis From Aortic Ring Explants

**Figure 5:**
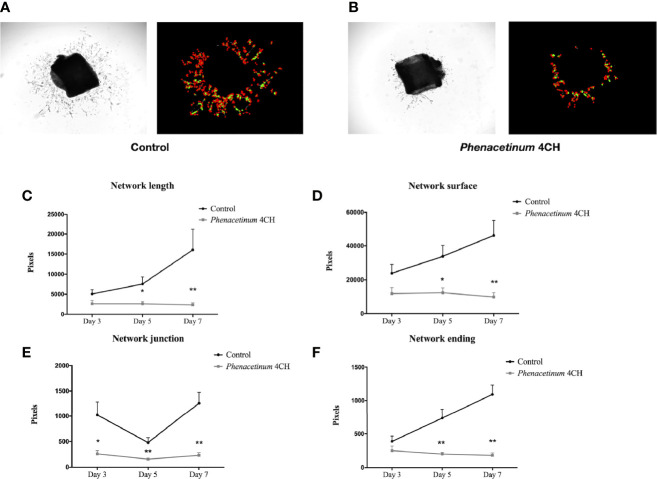
Effect of *Phenacetinum* 4CH on *ex vivo* aortic rings explants. Aorta were *ex vivo* seeded on Matrigel^®^. Phase contrast photos were taken at day 3, 5, and 7. **(A)** Representative photograph and modelling of control aortic assays using Image J software. **(B)** Representative photograph and modelling of *Phenacetinum* 4CH aortic assays using Image J software. **(C–F)** Semi-quantification at days 3, 5, and 7 of aortic rings network length **(C)**, surface **(D)**, junctions **(E)**, and ending **(F)**. (*p < 0.05, **p < 0.01).

In order to complete the study of the influence of *Phenacetinum* 4CH on tumor vascularization, we extended the study method on *ex vivo* aortic rings (**Figure 5**). The photographs and quantifications of the associated vascular networks (**Figures 5A, B**) show representative vascular budding from mice aortic rings, after 7 days of treatment by *Phenacetinum* 4CH or the control. Observations show clearly a decrease of the vascular growth with *Phenacetinum* 4CH treatment. A more detailed analysis of the quality of the network was performed using a plugin developed under Matlab. Analyses on day 3, day 5, and day 7 indicate that the length, area, junctions, and terminations of control mice vascular networks all increase over time (**Figures 5C–F**, black line). In contrast, treatment by *Phenacetinum* 4CH appears to clearly and significantly alter the growth and the quality of vascular networks. Indeed, when the length of the control networks triples in 4 days from 5077 pixels on day 3 to 16028 pixels on day 7, *Phenacetinum* 4CH network does not vary (2600 pixels), which gives a significant 65% inhibition on day 5 and 85% on day 7 (**Figure 5C**). This pattern is repeated for each of the characteristics studied. Concerning the network surface, it increases by 1.4 times every 3 days reaching about 46126 pixels on day 7 in control cases. Under conditions treated by *Phenacetinum* 4CH, the network surface is markedly decreased as a function of time, with 50% inhibition on day 3 and significantly on day 5 and day 7 with a 63% inhibition and 78% inhibition respectively (**Figure 5D**, grey line). The number of junctions in control case increases by 1.2 times between day 3 and day 7, but these junctions are significantly lower by 74% on day 3, 67% on day 5, and 82% on day 7 under the influence of *Phenacetinum* 4CH (**Figure 5E**). Finally, analysis of the vascular control network endings again indicated that the networks extend over time in the control rings (**Figure 5F**). There were 1.9 times more endings on day 5 than on day 3 and 1.5 times more on day 7 than at day 5 (**Figure 5F**, black line). Aortic rings treated by *Phenacetinum* 4CH showed 36% fewer endings on day 3 compared to the control rings, and significantly on day 5 with 73% fewer terminations and 84% on day 7. In conclusion, all these results demonstrate that *Phenacetinum* 4CH alters significantly the quality of the neoangiogenic network developed from the explants of aortic rings, early and sustainably.

### *Phenacetinum* 4CH Affects the Behavior of Endothelial Cells

**Figure 6:**
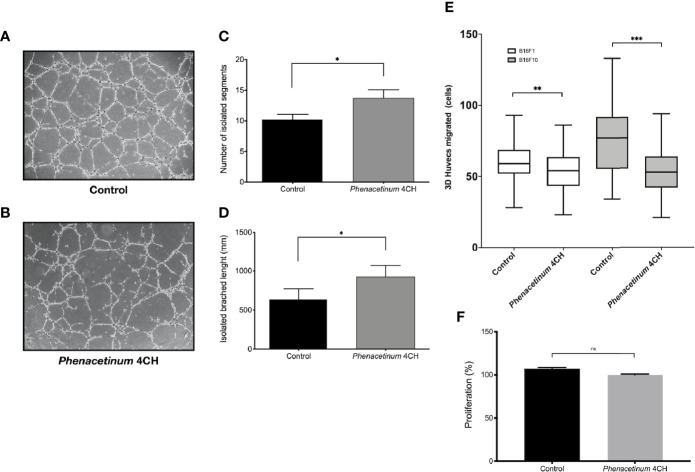
Effect of *Phenacetinum* 4CH on *in vitro* HUVEC tubulogenesis, migration, and proliferation. For tubulogenesis, HUVEC cells were seeded on Matrigel^®^. Phase contrast overview photos were taken after 6 h. For 3D migration, HUVEC cells were seeded on transwell coated with Collagen I and migrated cells on the lower membrane counted after 18 h. **(A)** Representative photo of control HUVEC tubulogenesis on Matrigel^®^. **(B)** Representative photo of *Phenacetinum* 4CH HUVEC tubulogenesis on Matrigel^®^. **(C)** Quantification of isolated segments. **(D)** Quantification of isolated branched length. **(E)** Quantification of HUVEC migrated cells with B16F1 (clear boxes) or B16F10 (gray boxes) conditioned media as chemoattractant. **(F)** Quantification of HUVEC proliferation. (ns, not significant, *p < 0.05, **p < 0.01, ***p < 0.001).

To reinforce these previous results and to demonstrate the potential anti-angiogenic effect of *Phenacetinum* 4CH, we realized tubulogenesis assays (34). After 4 h to 6 h of incubation, the vascular networks were photographed and analyzed using angiogenesis analyzer automatic plugin on ImageJ software. Photographs of tubulogenesis carried out under control conditions (**Figure 6A**) or under treatment with *Phenacetinum* 4CH (**Figure 6B**) are representative of the results obtained. The histograms represent the average number and the length of isolated segments after 6 h of incubation depending on conditions (**Figures 6C, D**). The cells treated with *Phenacetinum* 4CH induce 24.5% more isolated segments (13.9 on average) than the control cells (11.1 on average). Moreover, network splitting is 47.4% higher when HUVEC cells are treated with *Phenacetinum* 4CH compared to control cells (**Figure 6D**). Therefore, the *Phenacetinum* 4CH treatment damages the quality of the “pseudo” vascular network *in vitro*, increasing significantly the fragmentation of the branches and number of isolated segments. As migration is a critical step during angiogenesis, we performed 3D HUVEC cells transwell migration with B16F1 and B16F10 conditioned media as chemoattractant. As shown in **Figure 6E**, *Phenacetinum* 4CH inhibits 3D HUVEC cells migration in both conditions. Indeed, we observed an 8% and 31% decrease with B16F1 and B16F10 conditioned media respectively. By contrast, no change on HUVEC proliferation under *Phenacetinum* 4CH treatment was evidenced (**Figure 6F**), which strengthens the emerging view that *Phenacetinum* 4CH effects involve the migratory capacity of HUVEC.

### *Phenacetinum* 4CH Inhibits Melanoma Metastatic Dissemination

**Figure 7:**
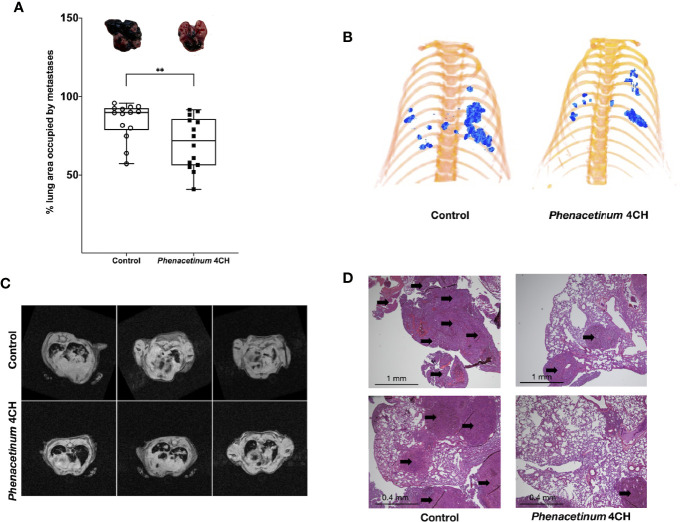
Effect of *Phenacetinum* 4CH on melanoma metastatic dissemination. 1 x 10^5^ melanoma B16F10 cells were injected in the lateral vein of C57BL/6 mice. Control (open circle) or *Phenacetinum* 4CH (filled box) (100 µl) were intraperitoneally injected each day. **(A)** Metastatic surface ratio (metastases/health lungs) at day 21 (median, n=10, Mann-Whitney test, **p < 0.01). **(B)** representative tridimensional reconstructions of mice at day 14 after µCT reconstruction. Metastases were segmented using Amira 6.5 software. Metastases are represented in blue. **(C)** T2 weighted images of the lungs from control (upper panel) and *Phenacetinum* 4CH-treated (lower panel) mice. Metastases appear as white opaque hyper-intense regions. **(D)** HPS staining of lungs section (left panel: control, right panel: *Phenacetinum* 4CH). Arrow identified metastatic foci.

As disseminated melanoma after tumor escape is a great therapeutic challenge and is subordinate to angiogenesis, we investigated the effects of *Phenacetinum* 4CH on an aggressive model of experimental metastases following intravenous injection of B16F10 melanoma cells in C57Bl/6 mice. In this model, systemic injection of *Phenacetinum* 4CH significantly decreases the surface of lung metastases on day 21 after melanoma cells inoculation (**Figure 7**). Lungs metastases were detectable on day 14 by µCT imaging with a differential development between mice treated or not by *Phenacetinum* 4CH (**Figure 7B**). Longitudinal monitoring until day 21 by RMI imaging highlighted an inhibition of the metastases development (**Figure 7C**). The total metastatic volume in the lungs was very large in the control mice (median volume ratio between metastatic and healthy surface lungs is 89%) compared to *Phenacetinum* 4 CH-treated mice (71% median volume ratio) as shown in **Figure 7A**. Histopathological analysis of lung sections confirmed the presence of large and numerous metastatic foci organized around blood vessels (**Figure 7D**). *Phenacetinum* 4CH treatment decreases significantly the presence of these foci. No side effects were observed in HES stain liver, kidney, heart, and spleen (**Figure S1**). These results highlight a potential anti-metastatic effect of *Phenacetinum* 4CH.

## Discussion

Over the past several decades, drug discovery has produced significant advances in metastatic cutaneous melanoma therapy. However, this remains a resistant and deadly disease where the angiogenesis plays a crucial role in the progression of solid tumor. Melanoma being considered a highly vascularized cancer, the use of adapted angiogenic inhibitors in therapeutic options is a relevant process to slow down its development (35–37). Previously, we demonstrated through an original work that homeopathic dilution *Phenacetinum* 4CH was able to exert anti-migratory properties on murine melanoma cell lines (22). In this work, we have evaluated the capacities of *Phenacetinum* 4CH to decrease the primary melanoma growth by disrupting tumor angiogenesis and evidenced that it may obviously reduce the metastatic progression. First, using a melanoma murine tumor model, we demonstrated that *Phenacetinum* 4CH delays early tumor growth and significantly improves the mice survival time. As a whole, these data highlight the antineoplastic potential of *Phenacetinum* 4CH on the primary melanoma. In a similar way, a natural molecule such as Baicalein has already shown an antitumor effect on animal tumor models, especially decreasing the cell proliferation and migration of B16F10 cells (38). Furthermore, Guillermo-Lagae et al. demonstrated that the antineoplastic impact of Honokiol (a phytochemical compound) observed *in vitro* on several melanoma cell lines was also checked in melanoma murine models (39). In our case, we demonstrated in a former *in vitro* study investigation that *Phenacetinum* 4CH did not affect the growth of B16 cells lines (22). Thus, we can assume that *Phenacetinum* 4CH does not act on the proliferative capacity of B16F1 tumor cells xenografts despite the tumor growth delay, implying another *in vivo* antitumor mechanism. Indeed, another study interestingly highlighted evidence that Curcumin intake, recognized for its anticancer properties, inhibits *in vivo* glioma-induced angiogenesis with the decrease of newly formed vessels (40).

More than 40 years ago, Judah Folkman revolutionized the way of thinking about the field of oncology (13). He postulated that, in order to survive and develop properly, tumors need nutrients and oxygen through a consequent blood supply, allowing them to evacuate metabolic waste and carbon dioxide (11, 12). Indeed, the center of the tumor can become quickly hypoxic and necrotic, thus altering its development. Based on these elements, angiogenesis clearly constitutes an interesting target for the cancer treatment. Therefore, it seemed relevant for us to understand the action of *Phenacetinum* 4CH on the neoangiogenesis of cutaneous melanoma. For this, we characterized the intra-tumoral vascular network by micro-computed tomography. A great number of descriptions showed that tumor vasculature is a chaotic mixture of deformed and enlarged vessels with a heterogeneous distribution within the tumor (41, 42). Here, we have demonstrated that mice treated with *Phenacetinum* 4CH show the same kind of vascular linearity deficiency, whose network seems to be limited to the periphery of the tumor, structurally more immature, and significantly less bulky than control mice. These data were confirmed by the analysis of histological sections of the tumors since *Phenacetinum* 4CH increased significantly the percentage of tumor hypoxia, and conversely, decreased the rate of blood vessels. Supported by literature, these data could indicate that under the action of *Phenacetinum* 4CH, blood vessels resulting from neoangiogenesis would be poorly structured and weakly disseminated within tumors, leading to a poor blood perfusion and an increase in hypoxic tumor areas (43–45). So, a defect in blood supply can be the cause of the delay in tumor growth.

Angiogenesis is governed by a set of molecular regulators that allow vascular extension and remodelling, in order to establish a mature and functional circulatory system to support tumor growth. To validate the hypothesis whereby *Phenacetinum* 4CH could act particularly on the structure of neoangiogenesis, we studied vascular budding from explants of mouse aortic rings. This technique was carried out in the presence of the VEGF growth factor in order to simulate the melanoma tumor microenvironment. Indeed, VEGF has been shown to be particularly necessary for chemotaxis, proliferation of endothelial cells, and their assembly into vascular and angiogenic structures (46). In these conditions, we have been able to demonstrate that *Phenacetinum* 4CH reduces early and durably *ex vivo* surface sprouting, elongation, and network branching. Moreover, *in vitro* endothelial cell tube formation assay suggests that *Phenacetinum* 4CH tends to alter the stability and quality of the capillary-like structure by increasing its fragmentation. Therefore, it would appear that *Phenacetinum* 4CH acts particularly on the spouting and/or the recruitment of endothelial cells, likely in a growth factors-dependent manner. Indeed, Rafii et al. reported in their review that initial phases of angiogenesis involve the endothelial cells recruitment on tumor site by two complementary mechanisms (47). One is based on a detachment of endothelial cells from preexisting vascular wall, and the other, on a direct recruitment of bone marrow-derived endothelial progenitor cells from the blood flow (10, 47). To appreciate this latest concept, our study was supplemented by a functional analysis of circulating micro-vessels using DCE-MRI in living organisms (48, 49). In addition to the usual information on the vascular network, it enabled us to evaluate the capacity of endothelial cells to be recruited within the plug under growth factors, and to quantify the progression of the vascularization (50, 51). Interestingly, the antiangiogenic activity of *Phenacetinum* 4CH was also shown by a significant reduction of the vascular density and haemorrhagic blood flow into the Matrigel^®^ plug. In the same way, other experiment advances that conditioned media of melanoma cells treated with *Phenacetinum* 4CH significantly reduced the HUVEC cells migration. Knowing the importance of the chemoattractant role of many growth factors and chemokines for the endothelial cells recruitment during angiogenesis, it makes sense to suggest that *Phenacetinum* 4CH impedes their secretion from tumor cells. Therefore, our results indicate a potential failure in the recruitment of endothelial cells in the angiogenesis process.

In the context of metastatic melanoma, resistance to anti-angiogenic therapies is set up through various mechanisms such as vascular mimicry, the vascular co-option, or bone marrow-derived vasculogenesis (10, 52, 53). All of these modes of tumor blood supply lead to the formation of a complex and dynamic microenvironment necessary for tumor survival. As mentioned above, the particular case of hypoxic tumor cells stimulates the recruitment and migration of vascular progenitor cells from the bone marrow that differentiate into endothelial cells or inflammatory cells infiltrating the tumor (54). Mostly, macrophages were able to polarize in response to inflammatory microenvironment and found themselves involved in many cancers, including melanoma, as an indicator of poor prognosis (55, 56). Among subpopulation of macrophages, the surface marker CD163 is usually targeted to identify tumor-associated macrophages (TAMs) (57). TAMs sense hypoxia in avascular areas of tumors and react in turn by releasing pro-angiogenic factors such as FGF-2, TNF-α, and VEGFs, which again promotes tumor oxygenation by the direct recruitment of endothelial cells (58, 59). Given that, increased CD163+ TAMs has been shown to be correlated with increased microvessel density and angiogenesis (60–62). Herein, the decrease of these CD163+ TAMs noticed within melanomas could be involved to limit the endothelial cell recruitment through decreased growth factors excretion, and finally explain the increase in tumor necrosis as observed under *Phenacetinum* 4CH treatment. This attractive result suggests that homeopathic treatment induces a strong disturbance of tumor macrophage infiltration that contributes to the reduced angiogenesis observed *in vivo*. Thus, we can assume that *Phenacetinum* 4CH treatment influences this vascular feedback, causing a disruption in the regulatory loop controlled by growth factors between the tumor cells, TAM and endothelial cells. Hence, a disturbance of the recruitment signals emitted from tumor cells, or a disturbance on the polarization plasticity of macrophages M2 depending on the presence of cytokines, could be involved (63). Moreover, a publication of interest has highlighted a surprising correlation between the plasma membrane fluidity of carcinoma cells with HIF1-α and VEGF rates. Thereby, a cholesterol depletion leading to lipid rafts disruption induced an HIF1-α upregulation and enhanced the VEGF secretion (64). Rather, our previous study showed that *Phenacetinum* 4CH induced a significant increase of plasma membranes cholesterol of B16 cells (22). The related increase of membrane stiffness could consistently have the opposite effect and prevent the secretion of specific molecules by B16F1 cells to limit TAM infiltration. In this sense, we have demonstrated that conditioned media of B16 cells treated with *Phenacetinum* 4CH really decrease HUVEC cells migration, indicating a perturbation of these factors release. All these indications mean that this treatment evidently plays a crucial role in modifying the membrane fluidity, disrupting the mutual cells communication and the tumor microenvironment composition, which in turn does not allow to stimulate angiogenesis.

Hence and positively, the discontinuity of this vascular feedback allows tumor growth to be limited by increasing the areas of hypoxia. Paradoxically, a hypoxic and acidic tumor environment constitutes a hostile microenvironment for tumor cells. It promotes the selection and clonal individualization of more malignant tumor cells, which acquire a mesenchymal phenotype, further encouraging metastatic spread (12, 65, 66). Through invasion and migration systems, the circulating tumor cells (CTCs) of the skin melanoma then invade neighboring tissues or organs through blood and lymphatic circulation (67). We know that *Phenacetinum* 4CH increases the hypoxic intra-tumor surfaces of melanoma *in vivo*, which could create a favorable environment to metastatic dissemination. In addition, metastatic dissemination is the most critical and dangerous feature of cutaneous melanoma (1, 5). We have shown that *Phenacetinum* 4CH treatment significantly reduced induced B16F10 metastasis. Moreover, in our previous study, we showed in a complementary way that *Phenacetinum* 4CH can limit the *in vitro* migration of isolated melanoma cells (22). We demonstrated that *Phenacetinum* 4CH causes a diminution of cell migration by a domino-like of mechanical alterations through a structural modification of the B16 cells plasma membrane. In this sense, we can suggest that *Phenacetinum* 4CH can also operate during the isolated tumor cells dissemination *in situ*, thereby attenuating metastatic spread induced by hypoxia into solid tumors. But it can also prevent the establishments of lung metastatic niches from circulating tumor cells, targeting critical stages such as immune escape, single-cell migration, and angiogenesis, which plays its nourishing function.

Cancer is also an inflammatory process where an aberrant induction of angiogenic pathways is closely linked to the metastasis development. Regarding the melanoma CTCs, they preferentially metastasize in the lungs, and strategically through the venules where the blood flow is reduced (68, 69). Furthermore, to reinitiate their growth and survive as macrometastasis, CTCs must develop partly neo-angiogenesis (70, 71). In this way, the importance of VEGF and TGF already known to induce the growth of cutaneous melanoma could also have an impact on the environmental tumor inflammatory effect (72). For example, the overexpression of these growth factors and chemokines (IL-4 and IL-10) alters the antitumor activity of dendritic cells and serves as chemoattractant to recruit pro-tumoral TAMs. Despite their influence on endothelial cell behavior, TAMs also have the particular immunosuppressive role to prevent tumor cell attack by natural killer and T cells during tumor progression (73). Conversely to this biological pathway, Davoodvandi et al. demonstrated the positive effect of a Resveratrol treatment in the mouse lung melanoma model, by an increase and reinforcement of these activated T cells (74). Interestingly, it turns out that resident macrophage population also participates to the formation of premetastatic niches, promoting extravasation of CTCs from capillary network on the secondary sites. Indeed, many scientific journals explain the particular relationship between cancer cell and macrophages, by which tissue factor releasing in ECM promotes cancer cell colonization and retention in the metastatic site (75–77). Closely linked with our previous data observed on primary tumor angiogenesis, complementary *in vivo* results disclose that *Phenacetinum* 4CH treatment decreases metastatic colonization of circulating B16F10 cells. In view of the fact this treatment prevents tumor inflammation by reason of a decrease of TAMs infiltration, this may explain, in part, the limitation of metastases noted within the ectopic tissue. Moreover, homeopathy has already been recognized as being able to reactivate the immune system in the context of a tumor in a pre-clinical study (78). Thus, *Phenacetinum* 4CH could legitimately have an impact in the mechanisms involved in immunity. Indeed, interestingly, Saha et al. demonstrated that different homeopathic dilutions of *Calcarea carbonica* targets the immune system of animals to reactivate the proliferation of T lymphocytes, previously inhibited by sarcoma cells, and induce tumor apoptosis (78). In the *in vitro* tumor context, the study of homeopathic treatment “Canova” is believed to be responsible for increasing leukocyte counts and T-cell proliferation (79). “Canova” in a sarcoma model shows that it can infiltrate lymphoid cells (Natural Killer lymphocytes) and have a tumor microenvironment cytotoxic action, which is a sign of favorable prognosis (80, 81). Finally, it has also been demonstrated that macrophages, in hypoxic tumor regions, express the ligands of inhibitory PD-1 and CTLA-4, which are already found themselves overexpressed in the malignant cutaneous melanoma and participate in tumor escape (73, 82). Therefore, assuming the *Phenacetinum* 4CH effect relies on the inflammatory component, as demonstrated previously with other homeopathic treatments, the joint use of immunotherapy (such as Ipilimumab or Nivolumab) with homeopathy could be a promising line of research. Indeed, a synergic action of this cotreatment type could then be set up to reinforce the cytotoxic effects of T cells against the tumor (79, 83, 84).

Collectively, all our results converge toward the promising therapeutic effect of *Phenacetinum* 4CH resulting in the slowdown of tumor growth caused by a decrease of angiogenesis. We were able to highlight some interesting effects like a deficiency in the migration and the recruitment of endothelial cells, an imbalance in the pro-tumoral macrophages despite hypoxia, and a structural malformation of the vascular network. All of these functions are governed by mutual interactions between cells, in particular through the secretion of cytokines and growth factors. Moreover, an evident reduction of lung metastasis is made sure through this treatment. Therefore, *Phenacetinum* 4CH can lead to fractures in the cyclic and chronic development of angiogenesis occurring naturally within tumors. Although the deciphering of the precise mechanism of the *Phenacetinum* 4CH effect deserves to be further considered, this treatment appears to have very exciting potentially complementary action mechanisms. The combination of these anti-angiogenic and anti-migratory actions placed in the complex tumor of cutaneous melanoma could constitute an innovative complementary treatment to existing cancer therapies. Thus, these various key functions involved in the metastatic melanoma development could enhance cancer therapy efficiency.

## Data Availability Statement

The raw data supporting the conclusions of this article will be made available by the authors, without undue reservation.

## Ethics Statement

The animal study was reviewed and approved by University of Reims Champagne-Ardenne (CEEA-RCA n°56) and the CNRS (Centre National de la Recherche Scientifique).

## Author Contributions

CF, SQ, CB, and CS performed the *in vivo* and *ex vivo* experiments. CF and ED managed *in vitro* experiments. E-HD and JD designed ImageJ and Matlab analysis. CB, SQ, CS, and JD analyzed µCT data. CS, CB, and JD performed and analyzed MRI experiments. CB-R and NB performed and analyzed IHC experiments. CF, LM, and CS designed research. CF and CS analyzed results. CF, SQ, ED, LM, and CS interpreted data. CF, LM, and CS contributed to write the manuscript. CF wrote the manuscript. LM and CS supervised the work. All authors contributed to the article and approved the submitted version.

## Funding

This research was supported by ANRT and Boiron laboratories.

## Conflict of Interest

The authors declare that the research was conducted in the absence of any commercial or financial relationships that could be construed as a potential conflict of interest.
