# Supplementary figures and images for: Anti-Tumoral and Anti-Angiogenic Effects of Low-Diluted Phenacetinum on Melanoma
Source: Front Oncol. 2021 Mar 3;11:597503. doi: 10.3389/fonc.2021.597503 (PMC7966719; doi:10.3389/fonc.2021.597503)

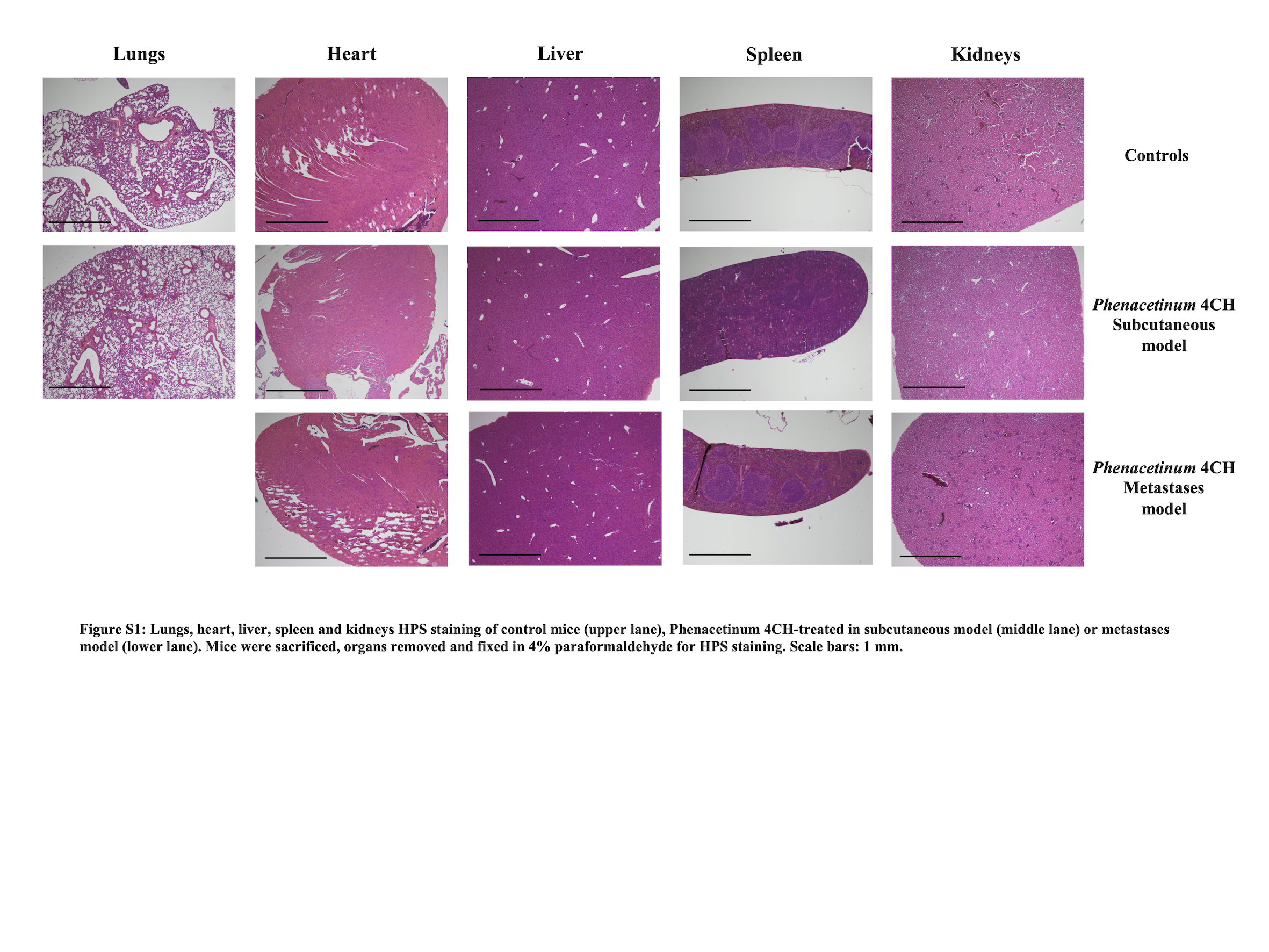

Supplement: Supplementary file 1 [file Image_1.tiff]
